# Cotton Woven Fabrics as Protective Polymer Materials against Solar Radiation in the Range of 210–1200 nm

**DOI:** 10.3390/polym15051310

**Published:** 2023-03-06

**Authors:** Polona Dobnik Dubrovski, Darinka Fakin, Alenka Ojstršek

**Affiliations:** 1 Mechanical Engineering Research Institute, Faculty of Mechanical Engineering, University of Maribor, 2000 Maribor, Slovenia; 2 Institute of Engineering Materials and Design, Faculty of Mechanical Engineering, University of Maribor, 2000 Maribor, Slovenia

**Keywords:** fabric engineering, solar protection, woven fabric structure, natural dyeing of polymer material, testing

## Abstract

The proposed paper describes the influence of woven fabric constructional parameters (type of weave, relative fabric density) and colouration (obtained by eco-friendly dyeing) on the solar transmittance of cotton woven fabrics in the range of 210–1200 nm. The cotton woven fabrics in their raw state were prepared according to Kienbaum’s setting theory, at three levels of relative fabric density and three levels of the weave factor, and then exposed to the dyeing process with natural dyestuffs (beetroot, walnut leaves). After ultraviolet/visible/near-infrared (UV/VIS/IRA) solar transmittance and reflection in the range of 210–1200 nm were recorded, the influence of the fabric construction and colouration were analysed. The guidelines for fabric constructor were proposed. The results show that the walnut-coloured satin samples at the third level of relative fabric density provide the best solar protection in the whole solar spectrum. All the tested eco-friendly dyed fabrics offer good solar protection, while only raw satin fabric at the third level of relative fabric density can be classified as solar protective material with even better protection in IRA region than some coloured samples.

## 1. Introduction

Woven fabrics are produced to fulfil different performance properties, which are essential for their application. In general, they should have adequate durability, a high level of comfort and aesthetic appeal, easy maintenance, support our health, and protect us against potentially hazardous substances [1]. During the last several decades, people have become more aware of the negative effects of excessive solar radiation on human health. According to the International Commission on Illumination (CIE), the spectral distribution of solar radiation at the surface of the Earth contains roughly 6.1% of ultraviolet light (consisting of 0.5% of ultraviolet radiation B (290–320 nm) and 5.6% of ultraviolet radiation A (320–400 nm)), 51.8% of visible light (400–780 nm), and 42.1% of infrared light (780 nm–1 mm) [2].

It has been reported that UV radiation not only has some negative effects on human health [3,4,5,6], but visible (VIS) and infrared (IR) radiation appear to have such effects as well, particularly the near-infrared radiation (IRA radiation, 760–1440 nm) [7,8,9,10]. The photo-ageing process is caused more by the IRA reaching the dermis than short-wavelength infrared (IRB) and mid- and long-wavelength infrared (IRC) radiation, which do not penetrate deeply into the skin [7].

We should also bear in mind that solar electromagnetic radiation transfers heat from the Sun to objects through space at long distances (thermal radiation). In the case of clothing, the transmitted thermal radiation through the fabric can heat the body (is absorbed) and influence thermophysiological clothing comfort. Since the ozone layer is depleting, the atmosphere is losing its natural protective capability, and therefore increasingly more solar rays reach the Earth’s surface. So, the clothing/fabric should possess adequate UV and thermal protection, allowing moisture to evaporate from the body into the environment simultaneously. At first glance, such a demand appears to be contradictory. Consequently, there is a growing need to develop an optimal fabric structure that could offer sufficient protection against solar radiation. First and foremost, the basic knowledge regarding the influence of fabric constructional parameters and finishing processes on solar protection properties should be fully understood in order to develop the material itself, which will then offer sufficient or optimal solar protection.

There are numerous kinds of research focused on the influence of woven fabric structure (raw material, type of yarn, type of weave, fabric mass, open porosity, tightness, etc.) [11,12,13,14,15,16,17] or the finishing processes [12,14,18,19,20,21,22,23] on ultraviolet protection. Yet less research is focused on the IR protection of woven fabrics. There are very few researchers who study the solar (UV, VIS, IR) protection of woven fabrics.

Yu et al. [24] studied the influence of fibre cross-sectional shape and fibre type on UV protection (reflectance and transmittance) of a single fibre/yarn and fibre bundle. They concluded that a triangular shape offers better UV protection. Zhang et al. [25] investigated the influence of woven fabric structure (fabric cover tightness, fabric thickness, area density) and temperature on IR transmittance (800–1400 nm) of the cotton and polyester woven fabrics. McFarland et al. [26] focused on the influence of moisture regain and fibre type on the IR absorption of woven fabrics.

Yildirim et al. [27] investigated the effects of weft yarn structure (fibre composition, yarn type, yarn linear density, yarn twist and hairiness, spinning method, and weft density) on plain woven fabric reflectance, transmittance, and absorbance in the UV, visible, and IR regions of solar radiation (282.5–2500 nm). The main conclusion was that by altering the yarn structure, the woven fabric transmittance was affected more in comparison to absorbance and reflection fabric properties. Jiang et al. [28] investigated the IR emissivity, reflection, and transmission rates, and Ultraviolet Protection Factor (UPF) of Ag-coated plain cotton and polyester woven fabrics in order to develop good UV and IR protection of tested samples.

In recent years, diverse natural colouring compounds (e.g., anthocyanin, betalain, naphthoquinone, carotenoid, flavonoid, aurone, chlorophyll, and indigotin) extracted from different parts of plants or other biological sources, have been proposed as UV protectors, when applied on textile materials (different type and forms), due to their low cost, biodegradability, low toxicity, non-carcinogenicity, etc. [29,30,31,32]. With the aim to enlarge the colour yield and affinity towards textiles (colour fastness properties), different natural or metal mordants are generally employed, thus influencing the materials’ UV transmittance ability and colour hue. High solar protection of naturally-dyed textiles depends on (besides the above-mentioned textiles’ constructional parameters) the chemical structure of the colouring compounds and their absorption characteristics in individual UV, VIS, or IRA regions [33], as well as the dyebath composition (liquor-to-weight ratio, pH, type, and concentration of mordant), and dyeing parameters (time, temperature) [29].

This study focused on the influence of the fabric structure (type of weave, relative fabric density, or tightness) and the eco-friendly dying on solar transmittance of cotton woven fabrics in the wavelength region between 210 and 1200 nm (UV, VIS, IRA). The woven samples were carefully engineered according to Kinebaum’s setting theory in order to achieve three levels of relative fabric density: 55–65% (minimum), 65–75% (average), and 75–85% (maximum). Two groups of samples were prepared: fabrics in a raw (grey) state (to produce environmentally friendly and healthy next-to-skin fabrics) and coloured fabrics, which were dyed with eco-friendly (natural) pigments extracted from red beetroot (*Beta vulgaris*) and fresh leaves of a common walnut tree (*Juglans regia*). The solar transmittance was measured using a UV/VIS/NIR test device and analysed to offer guidelines for fabric constructors by developing a cotton fabric with good/optimal solar protection.

## 2. Materials and Methods

The cotton woven fabrics were engineered according to Kienbaum’s setting theory and manufactured using the Picanol weaving machine under the same technological conditions. All woven fabrics were made from 100% cotton combed yarns with the following constructional parameters: fineness 25 tex, number of twists: 680 z, yarn diameter: 0.163 mm, volume coefficient: 4.564, bulk density of fibres: 1.5, yarn packing factor: 0.80, yarn flexibility factor: 0.8, yarn volume mass: 1.2 g cm^−3^. Firstly, the fabrics in a grey state were prepared (in order to eliminate the influence of finishing treatments on the solar radiation protective function of fabrics) at three levels regarding the type of weave (or weave factor); namely plain 10–01 01-01-00 (0.904), twill 20-02 02-01-01 (1.188), and satin 31-01 04-01-02 (1.379), and at three levels of relative fabric density: 55–65% (minimum, level I), 65–75% (average, level II), and 75–85% (maximum, level III). The warp (nominal) density of the fabrics was set to 29.2, 37.6, and 43.9 threads/cm for plain, twill, and satin fabrics, respectively. The weft (nominal) density of the fabrics was set between 13–23, 18–31, and 20–36 threads/cm for plain, twill, and satin fabrics, respectively. Afterwards, the warp and weft densities were measured again in accordance with the ISO 7211-2. Depending on different warp/weft densities of fabrics regarding the type of weave to achieve similar levels of relative fabric density, the fabric thicknesses were 0.350 mm, 0.400 mm, and 0.447 mm for plain, twill, and satin weaves, respectively. The basic fabric density was the same for all samples (5.605 threads per cm). The relative density of the fabric was calculated according to Equations (1)–(5):

(1) $$t=\sqrt{t_{1}\times t_{2}}$$

(2) $$t_{1}=\frac{G_{1}}{G_{lim}}\times 100\;\;\;\;\;\;\;\;\;\;\;\;\;\;\;\;\;\;\;\;\;\;\;\;\;\;\;t_{2}=\frac{G_{2}}{G_{lim}}\times 100\;\;\;$$

(3) $$G_{lim}=g\times V\times \sqrt{\frac{1000}{T}}$$

(4) $$g=5.117\times \sqrt{\rho_{fib}\times i}$$

(5) $$V=\frac{1.732\times R}{R+\frac{a\times \left(2.6-0.6\;z\right)}{f}\times 0.732}$$

where *t* is the relative fabric density or fabric tightness in percentages, *G* is the actual thread density in threads per cm, *G_lim_* is the limit density of fabric in threads per cm, *g* is the basic density of fabric in threads per cm, *V* is the weave factor, *T* is the yarn fineness in tex, *ρ_fib_* is the bulk density of fibres in g cm^−3^, *i* is the yarn packing factor, *R* is the number of threads in weave repeat, *a* is the number of double passages of yarn in one weave repeat (from face to back and vice versa), *z* is the smallest weave shift, and *f* is the yarn flexibility. Subscripts 1 and 2 denote warp and weft yarn, respectively. The constructional parameters of tested raw woven samples are listed in Table 1.

**Figure 1:**
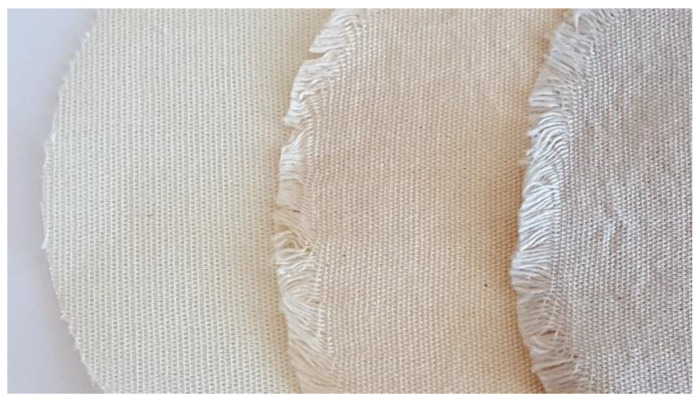
Photographs of raw and coloured tested cotton plain woven fabrics.

Woven samples in their raw states were further exposed to the dyeing process with natural dyestuffs in pastel colours to evaluate the influence of colouration on solar protection. The fabrics possessed three colour shades: beige (raw fabrics), light red (beet-coloured fabric), and light brown (walnut-coloured fabric) (Figure 1). The constructional parameters of the tested coloured woven samples are listed in Table 1. Before dyeing, the extraction process of cleaned beetroot (*Beta vulgaris*) and fresh leaves of a common walnut tree (*Juglans regia*), both chopped into small pieces, was completed with the aim of obtaining coloured compounds, i.e., betalains (dark purple–red) and juglone (dark orange–brown), respectively. Extraction of walnut leaves was accomplished inside boiling deionised (DI) water for 2 h [29], and extraction of beetroot peels inside DI water at a room temperature by means of a high-speed stirring for 4 h [34], using a liquor-to-weight ratio of 1:10. Both extraction mixtures were kept at room temperature for approximately 20 h, and then filtered for further use. Differently constructed fabrics were dyed according to the exhaustion procedure, employing the laboratory device Ahiba (Werner Mathis AG, Oberhasli, Switzerland), using a medium bath stirring method. Dyeing started at the temperature of 20 °C, where 30 g/L of NaCl and mordant (9 g/L of KAl(SO_4_)_2_·12 H_2_O for beetroot or 3 g/L of FeSO_4_ H_2_O for walnut) were added to the DI water, using a liquor-to-weight ratio of 1:100. The pH was adjusted to neutral (pH 7) by adding 1 mL/L NaOH (1 M). Afterwards, the dyebath’s temperature was gradually raised to 60 °C, with a heating rate of 3 °C/min. Then, 14 g of fabric was put into the extracted bath, which maintained circulation for 90 min at 60 °C. When dyeing was finished, the solution was left to cool down, and the fabric was gently removed from the bath, rinsed in warm and cold water, and dried at an ambient temperature. Prior to characterisation, coloured fabrics were conditioned under a standard atmosphere in a climatic room for 24 h at temperatures of 20 ± 2 °C and relative humidity of 65 ± 5%, according to standard ISO/R 139.

For measuring transmittance and reflection, a UV/VIS/NIR spectrophotometer Lambda 900 (Perkin Elmer, Waltham, MA, USA) was used in the wavelength range between 210 and 1200 nm in 10 nm intervals, and at a scanning speed of 450 nm per min. The device was equipped with a double-beam optical system and two detectors with an integrating sphere unit (60 mm with Spectralon coating), which is able to evaluate the total spectral transmittance of the scattering material. A photomultiplier tube (PMT) detector was used for the UV (and visible) region, and a low-temperature sulfide lead (PbS) detector for the NIR region.

The absorbance (*A*) was calculated from the transmittance (*T*) and reflection (*R*), according to the Beer–Lambert law for scattered samples using Equation (6):

(6) $$A=log_{10}\frac{1}{T+R}$$

## 3. Results and Discussion

### 3.1. The Effect of Type of Weave and Relative Fabric Density (Tightness) on Solar Transmittance

**Figure 2:**
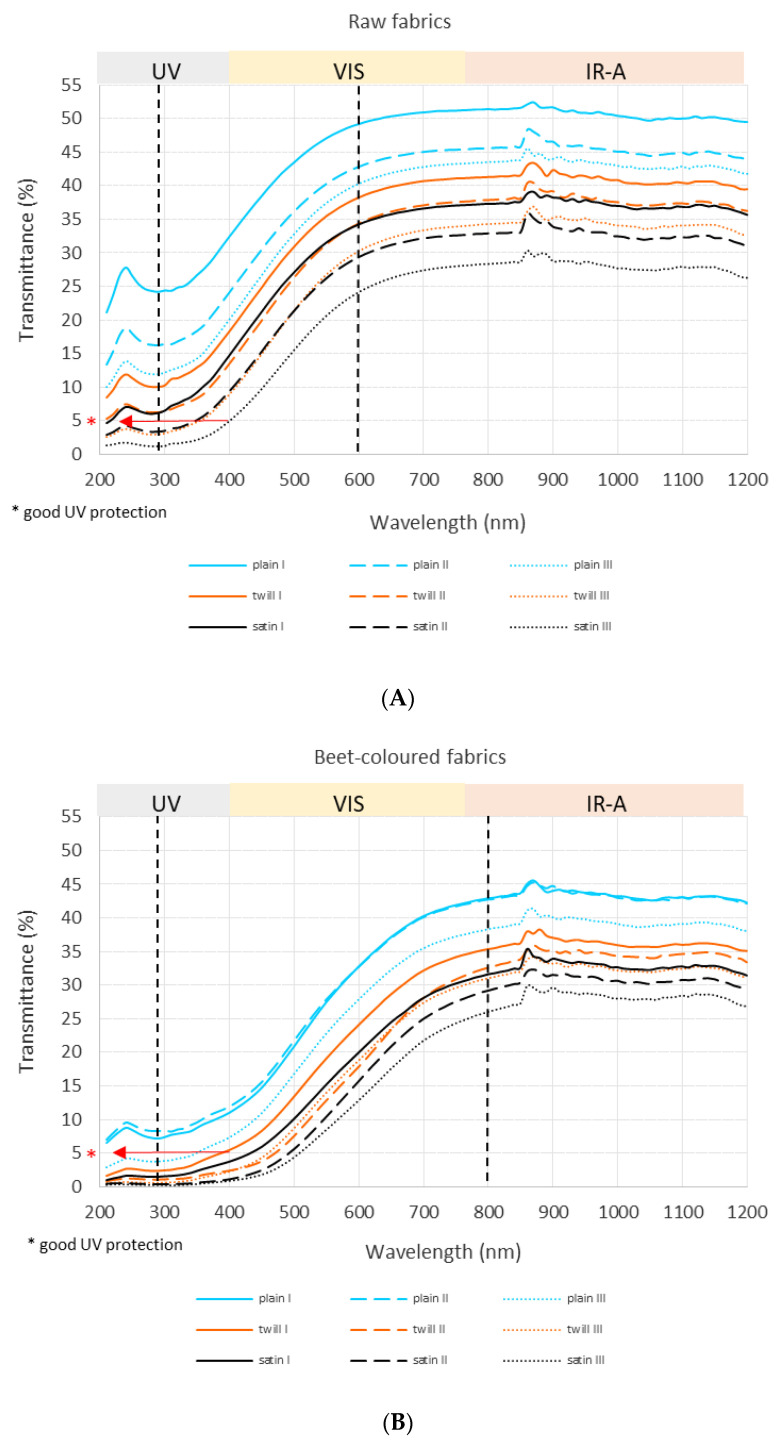

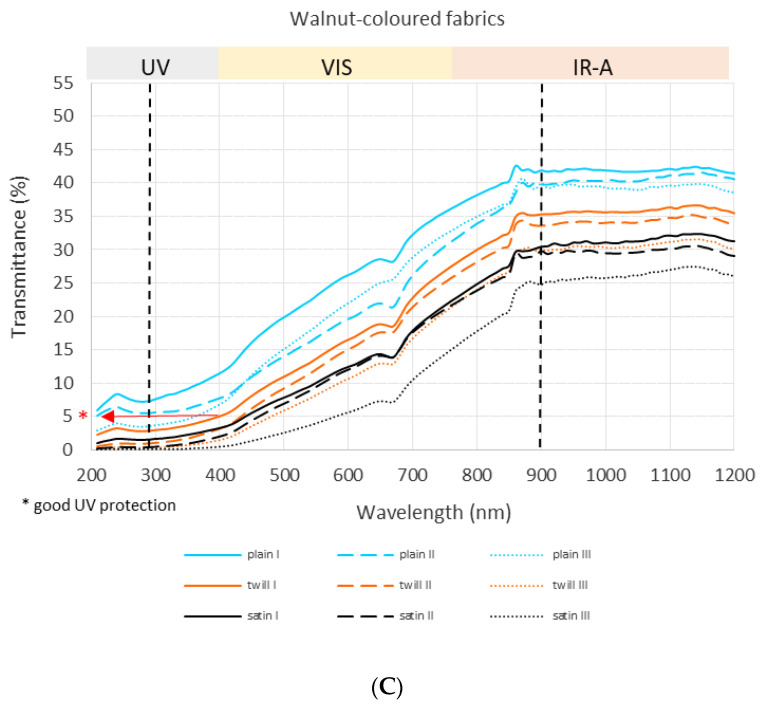
Transmittance curves of (**A**) raw; (**B**) beet-coloured; and (**C**) walnut-coloured cotton woven fabrics regarding the type of weave and relative fabric density.

The transmittance curves of the tested fabrics are shown in Figure 2. From Figure 2a, which refers to raw woven fabrics, three distinct regions of solar interaction with the fabrics in regard to wavelength can be observed: in the first two regions, which refer to wavelengths of 210–290 nm and 290–600 nm, the transmittance changed with the wavelength, while in the third region (from 600–1200 nm), it was very similar. In the region from 210–290 nm, first, the transmittance quickly increased together with the wavelength and then decreased, while the change in transmittance together with the wavelength in the region from 290–600 nm was almost linear (transmittance is higher at higher wavelengths). 

The transmission of UV radiation through the fabric was lower than the VIS and IR transmissions, depending on the fabric structure (valid also for beet- and walnut-coloured fabrics). The satin woven fabrics demonstrated the lowest transmittance of solar radiation through the fabric and offered higher solar protection, followed by twill and plain woven fabrics. It should be pointed out that woven fabrics were compared according to relative fabric density and not absolute warp/weft densities, and consequently, higher densities can be achieved in satin fabrics in comparison to twill and plain fabrics. Higher warp/weft density means lower fabric macroporosity, and thus a lower solar transmittance through the fabric’s macropores. Within each group of the type of weave, the samples with a higher relative density (higher nominal warp/weft density) also offered a higher solar protection. According to the EN 13758-2:2003 standard, the fabrics provide at least good UV protection if UVA and UVB radiation transmittance is less than 5% (denoted with the red arrow in Figure 2). The results indicate that within raw woven samples, only satin woven fabric at level III of relative fabric density can be classified as fabric with good UV protection. The highest values of transmittance of solar radiation for such raw satin woven fabrics were 4.2%, 28.1%, and 28.8% in UV (at 390 nm), VIS (at 770 nm), and IRA (at 840 nm) regions, respectively.

### 3.2. The Effect of Eco-Friendly Dyeing on Solar Transmittance

Figure 2b,c, which refer to coloured fabrics dyed with natural dyestuffs, also show three distinct regions of solar interaction with the fabrics in regard to the wavelength, but with slightly different limits compared to the raw woven fabrics. The boundaries of the first region (with the non-linear change of transmittance regarding wavelength) are the same as those by the raw woven fabric (210–290 nm), while the boundaries of the second region (almost linear change) are different (from 290–800 nm and 290–900 nm for beet-coloured and walnut-coloured fabrics (with a bit of depletion at 670 nm), respectively).

**Figure 3:**
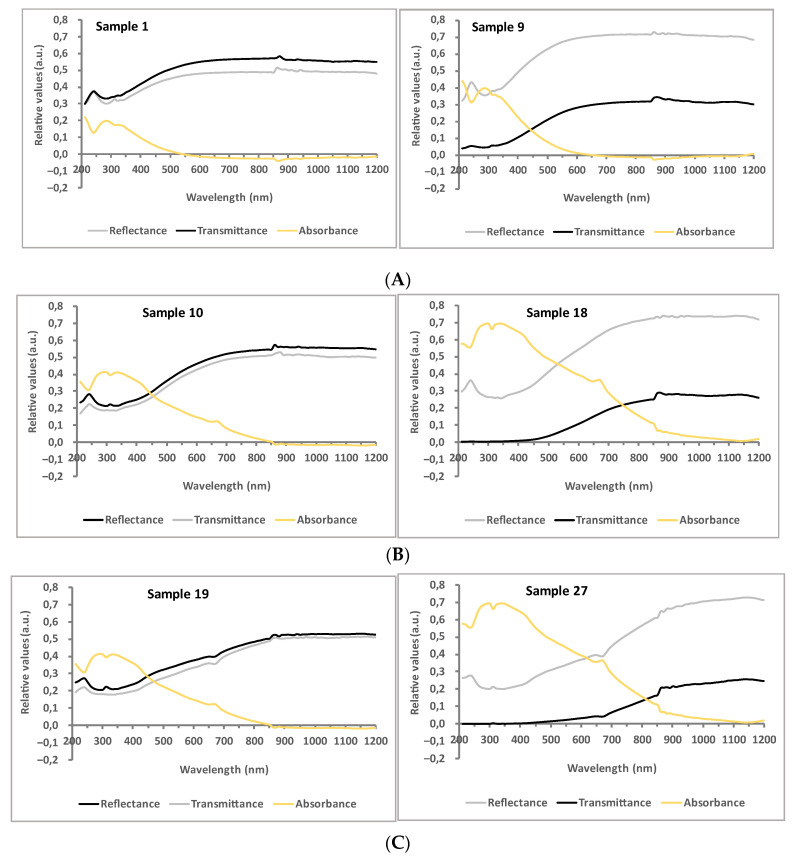
Relative absorbance, reflectance, and transmittance of plain fabric at level I of relative fabric density (**left**) and satin fabric at level III of relative fabric density (**right**): (**A**) raw, (**B**) beet-dyed, and (**C**) walnut-dyed.

Figure 3 compares the absorbance, reflectance, and transmittance of raw, beet-coloured and walnut-coloured samples at levels I (plain weave fabrics with the lowest cover factor) and III (satin weave fabrics with the highest cover factor) of relative fabric density, respectively. The reflectance and the transmittance were measured, while the absorbance was calculated according to Equation (6).

As can be observed from Figure 3a, the warp/weft density had a significant effect on the reflectance and absorbance characteristics of the fabric. High weft and warp density in the case of the satin fabric reflect more visible light compared to the plain weave fabric with a lower warp/weft density. In addition, higher warp/weft density caused an increase in absorbance, especially in the UV and VIS regions, due to the presence of a larger amount of natural pigments in the raw yarn. The application of natural colourants on the fabric surfaces, i.e., betalains in the case of beetroot dyeing (Figure 3b) and juglone in the case of walnut leaves dyeing (Figure 3c), additionally increased the absorption capability of the fabric compared to a raw cotton fabric (Figure 3a) within the same constructional parameters, lowering the transmittance curves in all three regions (as there is an inverse relation between transmittance and absorbance). Moreover, the visible absorption band shifted to a lower energy state upon complexation with metal ions due to the degree of chemical interactions, vibronic coupling, and changes in delocalisation of electrons between the fabric, mordant, and dye complex as explained in [33,35]. Obtained results indicate that juglone in combination with FeSO_4_ mordant (Fe-complex of the hydroxynaphthoquinone) had a broader absorption band compared to betalain (Al-complex of the cyclo-L-3,4-dihydroxyphenylalanine) as well as to natural pigments presented in the raw fabrics, widening the boundary in VIS towards IRA, as can be clearly seen from Figure 2 (from 600 nm towards 800 nm and 900 nm). The limits were defined at the average wavelength where absorbance actually became negative. In addition, the absorption band of both colourants is sensitive to the pH of dyebath; thus, the absorbance maximum of walnut leaf dyes is shifted towards higher wavelengths at pH 7 [29], while beetroot is shown to have a pale red colouration due to changed ratios of betaxanthin and betacyanin content in betalain [34].

In the case of naturally coloured woven fabrics, the results show that satin woven fabrics had the lowest transmittance of solar radiation through the fabric and offered higher solar protection, followed by twill and plain woven fabrics. Here, not only coloured satin woven fabric at level III of relative fabric density can be classified as fabric with at least good UV protection (with transmittance less than 5%) as in the case of the raw (beige) woven fabric, but also coloured satin and twill woven fabrics at all three levels of relative fabric density. For most samples (Table 2), the transmittance of UV radiation is smaller in walnut-coloured woven fabrics compared to beet-coloured fabrics. Samples (satin and twill) at level II of relative fabric density demonstrate the opposite result: the beet-coloured woven fabrics show a lower UV transmittance, although they were woven with similar warp/weft density. A closer look at walnut-coloured samples at level II of relative fabric density shows uneven dyeing on the surface with some lighter area, presumably due to the presence of unremoved greasy stains or impurities, which could influence the UV measurements. In VIS and IRA regions, the walnut-coloured samples (in all cases) show a lower transmittance in comparison to beet-coloured fabrics, and they offer better solar protection. While the coloured samples were compared at the same relative fabric density level and the weave type, one should expect they will have the same solar transmittance. However, we must remember that some solar radiation can directly pass through the fabrics’ macropores (direct transmittance), and some radiation is transmitted through the fibrous material, e.g., yarns (indirect transmittance). Here the colouration of the yarns plays an important role. 

More specifically, a higher or lower solar protection of dyed fabric is connected to the chemical structure of the colourant and its absorption properties in an individual spectral range [33]. As can be seen from Figure 2 and Figure 3, dyed fabrics had a lower transmittance compared to the raw fabric due to the high absorption ability of both natural dyes with absorption maximums in the UV/VIS region, and lesser absorption ability in the IRA region, meaning that IR radiation did not provide sufficient energy to the dyes, that could impart a π → π* transition of electrons within the dye chromophore molecules [36,37]. Feng et al. [38] proposed a UV-absorption mechanism and the photochemistry reactions of natural dyes, including tautomerism and chain reactions of free radicals upon UV light absorption. Moreover, the addition of Fe-based mordant caused a significant spectral shift of absorption bands towards the higher wavelengths in comparison to Al mordant, as mentioned above, thus influencing the transmittance of UV, VIS, or IRA light through the fabric [29,39]. Negative absorbance values at higher wavelengths in Figure 3 are influenced by the excess radiation (which was also reflected and transmitted) since fabric behaves as a black panel. Higher relative fabric densities caused a decrease in the emittance of the fabric. Similar results were reported by [27].

The highest values of transmittance of radiation for beet– and walnut–coloured fabrics with good UV, and thus, solar protection in UV, VIS, and IRA regions at individual wavelength are listed in Table 2.

**Figure 4:**
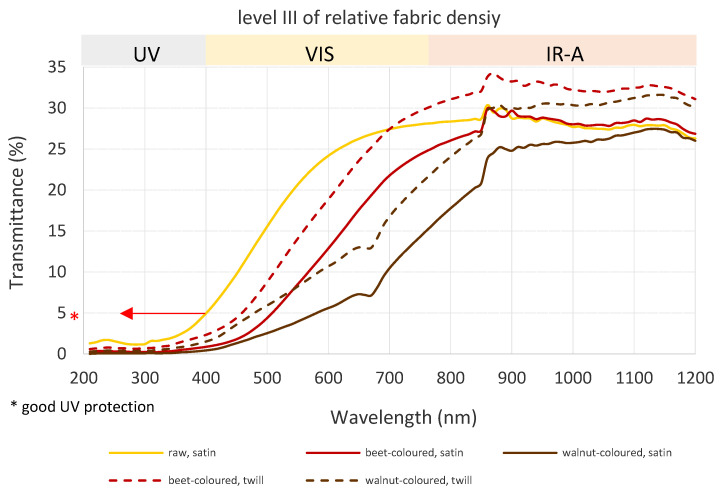
Transmittance curves of cotton woven fabrics at level III of relative fabric density with good solar protection.

Figure 4 shows the results of the transmittance of solar radiation through the cotton woven fabrics with the best solar protection at the third level of relative fabric density. From Table 2 and Figure 4, the following conclusions can be made, which may also serve as guidelines for fabric engineering for developing a cotton woven fabric structure with adequate solar protection:

- Taking into account the criteria that only those fabrics which transmitted less than 5% of UVA and UVB radiation offer good UV protection, the following samples can be classified as fabrics with adequate solar protection: all beet- and walnut-coloured samples, except plain-coloured woven samples, raw twill samples at level III of relative fabric density, and raw satin fabrics at levels II and III of relative fabric density;
- The walnut-coloured satin woven fabric at level III of relative fabric density provides the best solar protection with the lowest transmittance in all three regions (UV, VIS, IRA);
- Beet- and walnut-coloured twill samples at level III of relative fabric density offer better solar protection in comparison to beet- and walnut-coloured satin samples at level I of relative fabric density;
- Walnut-coloured twill woven fabrics at level III of relative fabric density provide better protection in the VIS region (from 550 nm) in comparison to beet-coloured satin woven fabrics at level III of relative fabric density, although the last-mentioned fabric is woven with a higher nominal warp/weft density. In contrast, within most of the IRA region, the beet-coloured satin woven fabric becomes more suitable as a protective material;
- Walnut-coloured satin samples at level III of relative fabric density (Figure 4) have the lowest transmittance, followed by beet-coloured and raw woven samples in the UV region. The difference in transmittance regarding the colour is more evident in the VIS region. In contrast, the difference is decreased in the IRA region, and from 1100 nm, there is only a slight difference between coloured samples;
- Depending on fabric structure, fabrics with light-shaded colours provide better IRA protection (and thermophysiological comfort). The IRA transmission of raw (beige) satin woven fabrics was lower than beet-coloured (in the whole region) and walnut-coloured (in most of the IRA region) twill fabrics.

## 4. Conclusions

The presented research indicates that the fabric structure and colour shades or colouration impact the fabric solar transmission (as well as reflection and absorbance) of cotton woven fabrics. It gives guidelines for planning the construction of woven cotton fabrics with optimal solar protection in the range of 210–1200 nm. While good UV protection does not always translate to adequate IRA protection, and thus better thermophysiological comfort, it would benefit woven constructors to have some prediction models of solar protection for different types of woven fabrics in order to predict solar transmittance, absorbance, as well as reflection in the whole UV–VIS–NIR range and consequently eliminate sample weaving, which is a topic for further investigation.

## Figures and Tables

**Table 1:** The constructional parameters of tested woven samples.

Fabric Code	Type of Material	Type of Weave	Warp Density (Ends/cm)	Weft Density (Pick/cm)	Level of Relative Fabric Density	Relative Fabric Density (%)
1	Raw	Plain	29.8	13.2	I	62
2	Raw	Plain	30.2	17.9	II	73
3	Raw	Plain	29.4	24.1	III	83
4	Raw	Twill	38.7	18.2	I	63
5	Raw	Twill	38.6	24.6	II	73
6	Raw	Twill	38.2	32.8	III	84
7	Raw	Satin	43.7	19.5	I	60
8	Raw	Satin	43.0	27.1	II	74
9	Raw	Satin	42.8	36.9	III	81
10	Beet-dyed	Plain	29.9	13.7	I	63
11	Beet-dyed	Plain	29.2	19.4	II	74
12	Beet-dyed	Plain	29.1	25.5	III	85
13	Beet-dyed	Twill	36.2	19.6	I	63
14	Beet-dyed	Twill	38.8	25.7	II	75
15	Beet-dyed	Twill	39.1	33.1	III	85
16	Beet-dyed	Satin	43.0	19.9	I	60
17	Beet-dyed	Satin	44.4	27.4	II	71
18	Beet-dyed	Satin	42.0	37.1	III	81
19	Walnut-dyed	Plain	29.6	13.6	I	63
20	Walnut-dyed	Plain	28.4	18.8	II	72
21	Walnut-dyed	Plain	29.0	26.1	III	86
22	Walnut-dyed	Twill	39.4	18.2	I	64
23	Walnut-dyed	Twill	39.0	25.7	II	75
24	Walnut-dyed	Twill	39.2	32.6	III	85
25	Walnut-dyed	Satin	45.2	19.8	I	61
26	Walnut-dyed	Satin	44.7	29.2	II	74
27	Walnut-dyed	Satin	41.6	37.1	III	80

**Table 2:** The maximum transmittance of solar radiation through the coloured fabrics, which offer good solar protection.

Fabric Type	Transmittance (%)					
	UV (390 nm)		VIS (770 nm)		IRA (950 nm)	
	BCF ^1^	WCF ^2^	BCF	WCF	BCF	WCF
Twill I	5.0	4.7	34.7	22.8	36.8	35.7
Twill II	2.2	2.9	31.6	21.4	34.8	34.1
Twill III	2.1	1.3	30.2	16.7	33.1	30.5
Satin I	3.4	3.1	30.8	17.9	33.3	31.0
Satin II	1.0	1.8	28.2	17.6	31.1	29.5
Satin III	0.8	0.3	25.1	10.4	28.8	25.6

^1^ Beet-coloured fabric; ^2^ Walnut-coloured fabric.

## Data Availability

The data that support the findings of this study are available from the corresponding author upon reasonable request.
